# Qualitative evaluation of the implementation of “Tuning in to Kids” in Norwegian Kindergartens

**DOI:** 10.1186/s40359-023-01088-4

**Published:** 2023-03-30

**Authors:** Vilde Randen Skåland, Sophie Seychelle Havighurst, Egil Nygaard, Inger Lise Teig

**Affiliations:** 1 Department of Psychology, University of Oslo, Oslo, Norway; 2 Department of Psychiatry, Mindful: Centre for Training and Research in Developmental Health, University of Melbourne, Melbourne, Australia; 3 Department of Global Public Health and Primary Care, University of Bergen, Bergen, Norway

**Keywords:** Tuning in to Kids for Kindergarten Teachers, Motivation, Implementation science, Emotion coaching

## Abstract

**Background:**

From January to June 2020, 22 FUS kindergartens across Norway implemented *Tuning in to Kids for Kindergarten Teachers* (TIK-KT) as part of a randomized control trial (RCT). Between the evaluation of an intervention and use of it in daily practice, a research-to-practice gap can often occur. The theory of planned behavior constituted the theoretical basis for the qualitative interviews that were administered to explore these gaps. This study aimed to explore motivation among kindergarten staff regarding the implementation of TIK-KT.

**Methods:**

Participants from the FUS kindergartens RCT were part of the current study. A stepwise deductive inductive strategy was used in the thematic content analysis. The data were from eleven semi-structured telephone interviews with kindergarten leaders and teachers. Codes from interviews before and after implementation were grouped based on thematic connections, and code groups were further combined into themes. The *Consolidated criteria for reporting qualitative research* were used as a reporting standard.

**Results:**

The interviews resulted in four main themes: (1) understanding the rationale of implementation, (2) "aha"-experiences, (3) the research-to-practice gap and (4) the main motivation. Kindergarten leaders and teachers expressed positive attitudes toward the intervention ideas and motivation to practice emotion coaching skills and toward implementing TIK-KT both before and after implementation.

**Conclusion:**

Kindergarten leaders’ and teachers’ motivation for implementation came from having a good understanding of the ideas of *Tuning in to Kids for Kindergarten Teachers* (TIK-KT), experiencing moments of “aha” regarding the intervention, not being held back by practical issues, and working toward their ultimate goal, the wellbeing of the children. These findings have implications for future implementation of TIK-KT and other mental health-promoting interventions and guide further areas of research to examine implementation mechanisms.

*Trial registration:* The study was registered with the Clinical Trials Registry (NCT03985124), June 13th, 2019.

**Supplementary Information:**

The online version contains supplementary material available at 10.1186/s40359-023-01088-4.

## Background

Findings from clinical research do not automatically translate into public health outcomes. Implementation science has developed to address the research-to-practice gap and can be defined as “the scientific study of methods to promote the systematic uptake of research findings and other evidence-based practices (EBP) into routine practice, and, hence, improve the quality and effectiveness of health services” [1]. Implementation studies typically evaluate the implementation process and its impact on the EBP of interest. In contrast, clinical trials typically focus on the health effects of EBPs and the question “does it work?”.

Stages-of-change theories describe the importance of awareness of and motivation for performing a specific behavior [2]. To understand what is embodied in “motivation”, the theory of planned behavior (TPB) was the theoretical guide in the present article. The TPB states that behavior is influenced by an individual’s intentions to perform a specific behavior, which in turn are influenced by attitudes (or motivations) toward the behavior, perceived social norms regarding the behavior and perceived control related to the behavior [2, 3]. In this paper, “motivation” is defined as “a process that influences the direction, persistence and vigor of goal-directed behavior” [4].

*Tuning in to Kids* (TIK) is an evidence-based program for parents and caregivers of children that aims to improve children’s emotional competence, behavior, and social functioning [5–7]. TIK-KT is a version of the program in which the focus is transferred from the parent/caregiver to kindergarten personnel. The program includes professional training in a group setting using role play and tasks to give teachers an understanding of children’s emotional competence and what promotes it. In the training, teachers learn skills in emotion coaching, a way of interacting by noticing a child’s emotion, connecting with the child, naming the emotion, empathizing and, if necessary, assisting with problem solving or setting limits on behavior [8]. The teachers are then supervised in their kindergarten to apply the emotion coaching skills in interactions with the children in their care, thereby providing ongoing opportunities to refine their use of the skills. An example of a similar program is Thrive by Three, a CLASS-based professional development program specifically aimed at increasing the process quality in toddler classrooms in Norwegian childcare centers. Buøen and colleagues did a RCT of Thrive by Three in 187 Norwegian kindergartens. Like TIK-KT, this is an in-service professional intervention to promote the quality of caregiver-toddler interactions. In their study, they concluded with the intervention having a positive effect on teacher-toddler interactions in both CLASS domains (Emotional and Behavioral Support and Engaged Support for Learning) [9].

The facilitation of the development of children’s emotional competence can further impact broader aspects of child functioning (behavior, social functioning, etc.) [5–7, 10–13]. While the positive effects of using these skills have an evidence base (i.e.: [5, 11, 14–16]), teaching them does not automatically guarantee that they will be applied in routine practice and ultimately improve the quality of care [17–20].

Implementation research seeks to understand and enhance the translation of research to practice within real-world conditions. Motivation is important in driving and giving direction to behavior. In this study, the aim was to explore kindergarten leaders’ and teachers’ motivation to implement TIK-KT. Qualitative semi-structured interviews were conducted before and after the intervention period. The following research questions were addressed: [1] What motivates kindergarten leaders and teachers to implement the intervention? [2] What are kindergarten leaders’ and teachers’ thoughts about any connection between their motivation and their application of emotion coaching skills to children? [3] How do kindergarten leaders’ and teachers’ motivation change from the start to the end of the intervention period?

## Methods

### Research team and reflexivity

The first author of this paper is a female graduate student in clinical psychology at the Department of Psychology, University of Oslo (UiO). She conducted the interviews. She had interview experience from earlier classes but no other experience or training in research settings. The research team, Professor Egil Nygaard at the Department of Psychology, UiO (Ph.D., Dr. Philos; male), and Associate Professor Inger Lise Teig at the Department of Global Public Health and Primary Care, University of Bergen (researcher II, Dr. Polit; female), also functioned as supervisors for the first author. Professor Sophie Havighurst at Mindful: Centre for Training and Research in Developmental Health, Department of Psychiatry, University of Melbourne (BA Hons, Dip Clin Psych, Ph.D.; female) is a program author of the TIK suite of programs and was the principal researcher on the TIK-KT intervention trial in Norwegian kindergartens.

A relationship was established with some of the participants prior to the study, as the interviewer attended the 2-day professional training in TIK-KT with kindergarten leaders and lead pedagogues. The participants knew the interviewer was a graduate student working on her thesis, which was a smaller study within Professor Havighurst’s main study on TIK in Norwegian kindergartens. The participants were informed of the interviewer’s interest in implementation science and curiosity about TIK-KT in kindergartens.

### Study design

From January to June 2020, 22 FUS kindergartens across Norway implemented TIK-KT as part of a randomized control trial. It was from these kindergartens that the participants for the current study were sampled. The methodological orientation underpinning the present study was thematic content analysis. The study used an in-depth qualitative stepwise deductive inductive (SDI) model to analyze the data [21, 22]. This model was a systematic approach with different stages in the research process. The raw data were generated through telephone interviews. All interviews were transcribed and analyzed to identify major themes. Because the SDI model has a generalizable understanding as its goal [22], these themes were tested as concepts. Between every stage, there was a test to check how the empirical material appeared from a more theoretical perspective. We used three of six such deductive tests, namely, the coding test, the grouping test, and the concept test.

#### Procedures

The TIK-KT intervention is a professional training for teachers. Kindergarten leaders and the lead pedagogue attended 2-day (14-hour) professional training in TIK to learn about emotion coaching and how to use it with children (December 2019). This training provided them with skills to supervise and support teachers (as resource persons) in their application of emotion coaching in their interactions with children. The training was provided in English by one of the program authors, Havighurst. The kindergarten teachers attended a one-day training (7 hours) on how to use emotion coaching and skills to understand and regulate their emotions (January/February 2020 in Oslo, Bergen, or Trondheim). This training was provided by three Norwegian TIK trainers, all with master’s or equivalent degrees in early education/clinical psychology. After the training day for teachers, the leaders or lead pedagogue conducted fortnightly supervision sessions with teachers at their kindergartens to practice emotion coaching and assist them in using the skills with the children in their care (January through May 2020). Kindergarten leaders or the lead pedagogue then received a half-day booster session with Norwegian TIK trainers to address issues and challenges they encountered in supporting teachers in using emotion coaching skills (March 2020). Structured manuals were provided for all kindergarten staff, and support material was also provided in an online format.

Participants were selected by convenience sampling. The first author of this paper attended the two-day professional training on TIK, provided information about the study, and invited to participation in interviews about the participants’ motivation for implementing TIK-KT. Six people were sampled, but three were not included due to lack of contact information and late responses. To interview a variety of kindergarten staff and to obtain more interviewees, a random selection of intervention kindergartens was called by telephone to invite others to participate. Those who accepted the invitation were given the plain language statement and consent via e-mail. Four persons gave their consent. From the seven interview subjects, six interviews preintervention and five interviews postintervention were obtained, with three missing interviews due to technical problems with audio recording. Apart from being leaders or teachers in one of 22 FUS kindergartens, the sample of interviewees varied regarding age, gender, education, and work experience. Further, out of the seven interviewees, three were kindergarten teachers, three were kindergarten leaders, and one was an appointed resource person with the task of supporting teachers. Of the four interviewees who participated at both interview times, three were kindergarten leaders, and one was a kindergarten teacher. Five of seven interviewees were employed at different kindergartens. One person was male, and six persons were female.

All interviews were conducted by telephone for convenience. The interviewees were at different sites across Norway, and there was only one interviewer. In addition, restrictions due to the COVID-19 pandemic made it difficult to meet in person. The interviews were conducted by the first author and audio recorded with a mobile phone application. The interviewees were encouraged to sit in a private area during the interview, but there was no other control of the presence of nonparticipants. The interviews were administered twice: in December 2019 before the one-day training of teachers (T1) and in June 2020 after six months of implementation (T2). The settings for data collection varied. For T1, most interviews were recorded in study rooms on the UiO campus, one was recorded in a nondriving car, and two were recorded in a quiet setting at home. For T2, all interviews were conducted in a quiet setting at home. A semi-structured interview guide was developed for both interview sessions. The interview guides had thematic and dynamic questions connected to the research questions, and the guides were based on the TPB (for example, “Would you say that there are any norms/attitudes in your kindergarten regarding starting/implementing new interventions?”). The interview guide for T1 differed according to the role of the informants (“teachers in your kindergarten”/”your colleagues”). None of the interviews lasted more than thirty minutes and field notes were used.All interviews were transcribed. Repeat interviews and returning transcripts for participants to check was not undertaken due to practical limitations. During the interviews, the participants mentioned many of the same themes and although we cannot be certain, data saturation may have been reached.

### Analysis

The first stage of the analysis was the coding of the transcribed interviews. Within SDI, the goals of coding are threefold. The first goal is extracting the essence of the empirical material. To achieve this, inductive coding should be grounded in the empirical data and correspond closely to interviewees’ statements to develop *empirically close codes (EC codes*). These codes are derived from the data (i.e., not from planned topics, theories, etc.) and could be terms present in the data material. The coding test questions whether the *only* way to derive the codes was from the specific empirical material [22]. The second goal is to reduce the volume of the material by sorting the codes into code groups. Codes that are thematically connected are grouped together, and irrelevant codes are sifted out [22]. Based on these code groups, we identified four major themes. Minor themes were not a part of our results. The third coding goal is to enable the generation of ideas. As we worked on the coding, we strove to encapsulate the nature of the interviewees’ statements. Since EC codes were developed on the basis of the interviewees’ statements, the generation of ideas was also based on details within the empirical data [22]. We did not include a coding tree due to the number of codes.

The conceptualization stage and the analytic process were driven less by empirical material and more by theory [22] as we explored associations between the major themes and theories and previous relevant research. When executing a concept test, we considered the extent to which emerging concepts—our findings in connection with relevant research and theory—were sufficiently abstract and thus more generalizable. In this study, we aimed to take our concept development into a form of generalization called *conceptual generalization,* a version of analytic generalization [22]. The data were coded by the first author of this paper. The major themes were derived from the data, and no software was used.

#### Ethics

For this study, approval was obtained from The University of Oslo Human Ethics Committee and the Norwegian Centre for Research Data (Ref #651181, December 2nd, 2019). All interviewees signed written informed consent forms and were guaranteed anonymity in reporting the results. Names have been changed to preserve confidentiality. Information about colleagues and children in kindergartens was handled with care and guaranteed anonymity. Data were stored in Services for Sensitive Data (TSD), a platform and a secure project area for public research institutions.

## Results

Eleven interviews were coded in their entirety and 222 codes were extracted, please see the supplementary information to see the extracted codes (Additional file 2: Tables S1 and S2). Codes with the same wording or meaning were collated, resulting in sixty-one codes from T1 and eighty-three codes from T2. These were sorted into twelve code groups with four main themes (Additional file 2: Tables S3 and S4): [1] understanding the rationale, [2] aha experiences, [3] the research-to-practice gap and [4] the main motivation. In the following sections, the main themes will be described, and excerpts of interview responses will be provided to illustrate these themes. The interview excerpts were translated from Norwegian by the first author. The *Consolidated criteria for reporting qualitative research* were used as reporting standards; please see Additional file 1 for more details.

### Understanding the rationale of implementation

This theme emerged early in the coding process. It was often mentioned, and every interviewee attributed great importance to it: the importance of understanding *why* one ought to do something, of obtaining a good understanding of what was wanted and what the program entailed and of a a deep and *mutual* understanding were critical to implementation. For example, one of the leaders described how the rest of the childcare workers responded at the beginning of the implementation:

Anne, T1:For some it’s experienced somewhat as an *effort*, and slightly like, “Oh, is there *even more* that is new […] when we kind of have *passed* that, and it’s been introduced… I don’t experience that at all then. […] I experience them as *enthusiastic,* and they are *interested*….

Anne noted that teachers thought that starting a new program might be overwhelming, but as they gradually *understood* more of what the program entailed, it seemed more manageable and even sparked interest and motivation.

Anne, T1:There is nothing that is *controversial* here; these are things that we… agree with, entirely […] and that might also make it *easier*. To think that *this* is something we can do over time, this is something we agree with.

The interviewees recognized several aspects of the program and experienced these as helpful. The following excerpt from Anne (T1) is representative of this idea:

In one kindergarten, the leader and resource person spoke excitedly about the training days but subsequently found that the rest of the kindergarten teachers were disappointed after the one-day training in TIK-KT. To explain their excitement, they made their own introduction and emphasized, “It was a lot about *why* TIK is important, why it is so important to acknowledge children’s emotions” (Marte, T2). In other words, they explained the rationale behind TIK-KT and the implementation and the things they were excited about. Marte described the situation before and after her colleagues received an extra presentation on the TIK-KT intervention:

Marte, T2:Then, they said, “Now we understand, now we understand what she said, the other woman” [referring to the one-day training provided by a Norwegian TIK trainer]. […] Because they had not entirely understood it on the training day, gone through it slightly fast (…) However, after that, it became so much better. We started with supervision quite fast and managed it, and… truly… the progress for the personnel just exploded from there.

Marte describes how her colleagues’ disappointment changed: when they understood TIK-KT in greater depth, they started utilizing it. The importance of change was highlighted by the informants, as the following dialog reveals:

Anne, T2:You must create a good understanding for the others. Because otherwise it will, uhm, *definitely* not come to a change.

Interviewer:What do you think the employees think of their own *ability* or *control* to… change their ways of working?

Astrid, T1:No, I think […] make everybody understand *why* … then that process will be quite ok!

Additionally, the research behind TIK-KT was emphasized by the interviewees as an important reason for them to use the intervention. Karianne outlines this concept:

Karianne (T1):[…] this is not something that one has just *come up with*, that this is what we are going for, it is because it is professional… It’s science.

The interviewees wanted to obtain a sufficiently deep understanding of what the intervention entailed—theoretically and practically—to implement it. The TIK program is evidence-based, and this in itself was an important motivator for why they should put effort into delivery.

### Aha experience

Three interviewees spoke of aha experiences as a change in perspective or raising consciousness about something they had been doing. Astrid (T2) described how several of her colleagues experienced “aha”:

Astrid:And some people did have such aha experiences in between that this actually works. (…) And that is… a good starting point.

Interviewer:Yes. Do you think your colleagues seemed motivated?

Astrid:Yes, absolutely!

In contexts where kindergarten personnel experienced aha moments when practicing TIK-KT, they reported back to Astrid that it was effective. Kristine and Janne also mentioned aha experiences:

Kristine (T2):Therefore, for me it became slightly like an aha experience about… now I kind of work the way I think I *wish* to do it, being so close.

Janne (T1):(…) That is how *you* do it… However, maybe you should not be doing it any longer, so, to get a little of that aha experience that I think is quite important when you’ve worked in a kindergarten for so many years.

We can see that the interviewees may have developed a habitual way of working in which they may not reflect on the best way of doing things. When they experience a contrast to what they normally do, i.e.,an aha experience, changes to their practices are likely to occur.

### Research-to-practice gap

All interviewees mentioned a variety of practical challenges they found important when implementing the new program in their kindergarten, including time use, meeting places, the same information for all teachers and the focus of the tasks. These constitute the third main theme from the interviews. Thomas explained why he and his section wanted to continue to use TIK-KT in the future:

Thomas (T2):I think it’s a nice tool to have because now everyone’s familiar with the tool… And then TIK is concrete to the extent that it’s easy to use […], which makes it easy to connect to situations in daily practice […].

Karianne, (T1):Therefore, I imagine that it, it depends a little on time, truly […]. That one gets to use *enough time* on this. […] And kind of that … it’s *much* better that one spends time and *gets the hang of it* than to kind of rush past it and then *kind of* understand it.

A practical challenge many mentioned was how to meet with their colleagues at planned meetings, and Astrid (T1) called it “the main *obstacle*” to make things happen in kindergarten. Having such undisturbed meeting points for discussions and talks was important, as Karianne puts it:

The interviewees suggested that learning to do something new takes time and that practicing TIK-KT means that they have to slow down and stop when emotion coaching. Without stopping, teachers were less likely to implement the TIK-KT ideas. Anne described the research-to-practice gap and reflected on what might be the cause:

Anne (T1):[…] I can… have a feeling that I have the understanding, but still I don’t see the change in practice. […] Uhm, change in practice is *difficult* to achieve… And I think it’s a great deal about… uhm, having energy excess to follow it up over time.

Anne (T2):Then, it happens what so often happens in kindergarten, with illness and people whom, uhm, we might have to help out at other sections on a day, and then the focus kind of disappears a little, so then we have more of a “make the day go around” kind of activity.

Other interviewees had similar experiences of this lack of sufficient personnel. They emphasized that kindergartens have a legal requirement for a minimum number of childcare workers to avoid overloading those present with tasks and responsibilities.

### The main motivation

The fourth main theme was explicitly mentioned by most of the interviewees: their main motivation was acting in the best interests of the children. From the theme “understanding the rationale of implementation”, it is clear that knowing why one ought to do something and having a mutual understanding among colleagues were important to motivation. In “the main motivation”, the interviewees answered why they did things and where their mutual understanding lay: in what is best for the children. The following excerpts highlight this:

Samira, T1:We do, after all, want the best for the children who attend this kindergarten.

Astrid, T2:… the main motivation is that the children shall have it …. as *good as possible*, that we shall be *the best* grown-ups for children in our kindergarten. […] that is a good *motivation*…

By using “we”, they point to the sense of common understanding. In sum, the teachers and leaders described the belief that the tasks and expectations were for the greater good of the children in their care as motivating at both T1 and T2.

## Discussion

This study aimed to explore participants’ motivation for the implementation of TIK-KT. In the following sections, we first clarify how we understand “motivation”. Thereafter, we discuss our findings in relation to our research questions.

### How do we understand “motivation”?

In this paper, “motivation” is defined as “a process that influences the direction, persistence and vigor of goal-directed behavior” [4]. The theory of planned behavior (TPB, 3) is a well-known and well-studied cognitive theory of behavior change that focuses specifically on “a motivation to change” [2], and this theory was the foundation for the questions that were asked in the interviews. According to the TPB, what determines intentions and actions is salient information, or beliefs, relevant to the behavior. These salient beliefs are the antecedents of attitudes, subjective norms, and perceived behavioral control. The antecedent to attitudes is *behavioral beliefs* that influence attitudes toward the behavior, whereas *normative beliefs* are the antecedent to subjective norms and *control beliefs* are antecedents to perceptions of behavioral control [3].

### What did we find?

Based on our interviews, understanding the rationale of implementation was critical for motivation. If this understanding was lacking, kindergarten leaders were more likely to experience resistance. A better understanding of the intervention reduced resistance and increased motivation by making the intervention more manageable and interesting. Kindergarten leaders and teachers had beliefs about TIK-KT, for example, that it would be in the best interest of the children. To have beliefs about TIK-KT, they needed to know which practices and ideas the intervention entailed*.* In other words, because salient beliefs are antecedents to behavior, understanding and knowledge play a role. This was most certainly in accordance with the interview subjects’ opinions.

Some interviewees spoke on behalf of their kindergartens when stating their common motivation and goal: what was best for the children. Those who regarded the intervention to work toward this goal found this belief motivating and important when implementing TIK-KT. The interviewees emphasized the scientific value in the context of understanding and knowledge, and they appeared to regard research-based interventions as the most potent way to maximize children’s wellbeing. TIK-KT’s empirical foundation was an important rationale for why kindergarten staff put effort into its delivery.

All interviewees mentioned a variety of practical challenges that impacted the implementation of the new intervention in their kindergarten, i.e., time constraints and sufficient personnel. These practical challenges are likely to apply to a range of workplaces. Nevertheless, the challenges mentioned were repeated by several individuals. Hence, we saw this “package” of challenges as specific to the system they were in: the kindergartens implementing TIK-KT. Our conclusion was that their motivation for applying the emotion coaching skills to children would matter less if they felt hindered by practical issues.

To summarize, we believe that when kindergarten leaders and teachers, as well as people in general understand and presumably agree with the reasons for an intervention, these individuals are more likely to be motivated to make the effort and take the time to learn and practice new skills and behaviors. Learning, practicing and understanding *how* to use intervention-related skills increase the potential for efficient and successful use of the intervention, which in turn can strengthen positive beliefs and boost motivation. Strong beliefs that something will not be useful can hinder learning and the application of new skills. For some teachers, this was a theme. The challenge, therefore, was how to address these beliefs so that they would not impede the effective implementation of the emotion coaching skills.

An aha experience is not the same as understanding the rationale of implementation. “Understanding” can be defined as achieving “a grasp of the nature, significance, or explanation of something” [23]. The aha experience is a hallmark of sudden insight. Reber and Skaar [24] describe such experiences as having four defining features: [1] a sudden insight leading to a change in [2] processing fluency that increases [3] positive affect and [4] certainty that the insight is true. Experiencing “aha” leaves a person with positive affect and *subjective* certainty (assumed knowledge, but not necessarily fully understood yet) about the newly acquired insight, which seems to facilitate motivation. As Liljedahl [25] writes, beliefs, attitudes and emotions are generally understood as composing the affective domain. Attitudes and beliefs are stable entities, while emotions are relatively unstable. He suggests *changing emotions* to achieve changes in beliefs and attitudes. The role of the affective domain and the differing nature of the aha experience and understanding in general could potentially be key components of motivation and are worth exploring in the context of learning new interventions.

Among the interviewees, one personally experienced an aha moment, another was told about it by a childcare worker during the implementation of TIK-KT, and a third mentioned it as an important experience to reflect upon one’s work practices. They all spoke positively of the aha experience and understood it as important for change. Reber and Skaar [24] concluded in their study that an increase in metacognitive feelings (processing fluency, positive affect, and certainty) is very important in understanding the role of the aha experience in motivation and coping. In the current study, we interpreted our results as suggesting that people were motivated by a degree of positive affect, such as enthusiasm and excitement, coupled with understanding the ideas.

### Our findings in relation to prior research

Luo, Reichow [26] conducted a systematic review and meta-analysis of classroom-wide social-emotional interventions for preschool children. They found improvements in social and emotional competence as well as decreases in challenging behavior. From this, they drew support for the use of comprehensive social-emotional interventions “for all children in a preschool classroom to improve their social-emotional competence and reduce challenging behavior” (p.1). Our findings indicate that kindergarten staff are motivated for TIK-KT, a classroom-wide social-emotional intervention. The Norwegian Institute of Public Health’s 2018 report on children and youth mental health [27] called for more Norwegian research regarding the types of interventions that can be preventive and health-promoting for children in kindergarten. Regarding the kindergarten as an arena for intervention, there are very few evaluations of preventive interventions compared to schools. Nevertheless, kindergartens are optimal places for delivering social-emotional interventions that promote development and prevent difficulties while also having positive impacts on the social, emotional, and behavioral outcomes of preschool children (i.e.: [6, 26, 28]). However, this requires high-quality kindergartens that have structural quality (i.e., group size), process quality (i.e., listening) and result quality (i.e., children’s well-being) [29]. Our study adds to research on interventions in Norwegian kindergartens.

The literature on implementation science adds to the growing evidence that multifaceted or blended implementation strategies are necessary to enhance the quality and effectiveness of interventions, but the understanding of how and why such strategies work is poor [30]. This is unfortunate because it contributes to the assumption that “more strategies will achieve better results” (p.2) and the use of more resources than necessary. Lewis and colleagues argue that knowledge of implementation mechanisms (processes or events through which an implementation strategy operates to achieve desired implementation outcomes) would help to create more efficient implementation strategies that fit the specific contextual challenges. By exploring the motivation for implementation in a specific context, our study, supported by other research, may contribute information that can be developed in the possible generation of hypotheses and further development in this research area. We explored the construct of motivation to gain in-depth information about the plausibility of the construct to account for behavior change, thereby adding specificity.

Leeman et al. [31] conducted a survey evaluating the factors that influence the use of school health implementation tools and gained insight into how the tools were used and why their use was varied. The two frameworks guiding the evaluation were the Interactive Systems Framework (ISF) for Dissemination and Implementation and the Consolidated Framework for Implementation Research (CFIR). Overall, their findings on what tools were valued align with our findings on important themes regarding motivation for implementation. The school staff’s use of emotion coaching was influenced by contextual factors within three CFIR domains: the inner setting, the outer setting and the characteristics of individuals. Regarding the characteristics of individuals, they identified knowledge, beliefs and self-efficacy as themes in addition to school staff motivation. Access to knowledge and information and teachers’ access to professional development related to the tools were among the themes in readiness for implementation (inner setting). Regarding the characteristics of the tools that influenced their use by support systems staff, credibility, compatibility, and complexity were important. Likewise, our findings indicate the importance of knowledge, information, and professional development. Additionally, motivation in the form of aha experiences and what was seen as best for the children aligned with the more general motivation participants frequently mentioned in the evaluation by Leeman and colleagues. Our theme of the *research-to-practice gap* involves kindergarten leaders’ and teachers’ experience of practical issues. Such practical issues were included in the *compatibility* theme in Leeman et al.’s survey.

### Strengths and weaknesses

The data for this paper were gathered by the first author. One strength of the paper is that internal consistency was high regarding the transcription and coding of the interviews. Conversely, this could be a weakness in that the interpretations and perspectives were limited to those of one person. Missing interviews were not repeated due to limitations in work capacity. However, it was never both the T1 and T2 interviews with the same participant that were missing. Therefore, the person’s perspectives and opinions were still included, but in a general sense. The first author attended the training on TIK-KT; hence, she was attached to the ideas, and it was therefore possible that she may have been biased in her views of what the interviewees reported. To reduce these weaknesses, the coauthors provided feedback and discussion on the content and process. The first author was supervised by Inger Lise Teig regarding the coding to reduce bias. Another strength of this paper was the opportunity to explore kindergarten leaders’ and teachers’ experiences in depth because the qualitative approach resulted in rich data. Furthermore, this approach made it possible to grasp how the interviewees’ descriptions changed both during a single interview and between interviews at two different times. However, we can only reflect on and understand what the interviewees themselves discussed with respect to their practices. We were unable to directly measure what they did, so there may be a mismatch. We aimed to make this paper transparent in the sense of being explicit regarding the authors’ interpretations.

## Conclusion

Kindergarten leaders and teachers expressed positive attitudes toward the intervention ideas and motivation to practice emotion coaching skills and to implement TIK-KT at both T1 and T2. Their motivation came from having a good understanding the rationale of implementation, aha experiences regarding the intervention, not being held back by practical issues, and working toward the children’s wellbeing. The findings of the present study add to the knowledge base about enablers and barriers to the implementation of this type of intervention. Further, this knowledge enhances insights into the participants’ experience of implementation and provides valuable insights for the possible development of implementation strategies for the TIK-KT intervention. Future research may investigate potential causal pathways between motivational factors and implementation fidelity to enhance the understanding of implementation mechanisms and consider the possible advantages in developing an implementation strategy for the TIK-KT intervention.

## Supplementary Information

**Additional file 1**.** Adherence to Reporting Guidelines.** The* Consolidated criteria for reporting qualitative research* (COREQ; [32]) was used as reporting standard for this paper. COREQ is a 32-item checklist for interviews and focus groups.

**Additional file 2**.** Table 1**. All codes Time 1.** Table 2**. All codes Time 2.** Table 3**. Code groups.** Table 4**. Themes.

## Data Availability

Anonymized datasets generated and analyzed during the current study are not publicly available due to the qualitative nature of the datasets which are, despite anonymization, in their entirety potentially too sensitive for publicly availability. However, anonymized data is available from the corresponding author on reasonable request.

## Abbreviations

CFIR: Consolidated framework for implementation research
EBP: Evidence-based practice
EC codes: Empirically close codes
ISF: Interactive systems framework
SDI: Stepwise-deductive inductive
TIK: Tuning in to Kids
TIK-KT: Tuning in to Kids for Kindergarten Teachers
TPB: Theory of planned behavior
TSD: Services for sensitive data
T1: Time one
T2: Time two
UiO: University of Oslo
