# Supplementary material for: Qualitative evaluation of the implementation of “Tuning in to Kids” in Norwegian Kindergartens
Source: BMC Psychol. 2023 Mar 30;11:87. doi: 10.1186/s40359-023-01088-4 (PMC10060915; doi:10.1186/s40359-023-01088-4)
Supplement: Supplementary file 1 — Additional file 1. Adherence to Reporting Guidelines. The Consolidated criteria for reporting qualitative research (COREQ; [32]) was used as reporting standard for this paper. COREQ is a 32-item checklist for interviews and focus groups. [file 40359_2023_1088_MOESM1_ESM.docx]

**Adherence to Reporting Guidelines**

The *Consolidated criteria for reporting qualitative research* (COREQ; Tong, Sainsbury, & Craig, 2007) was used as reporting standard on the paper *Qualitative evaluation of the implementation of "Tuning in to Kids" in Norwegian kindergartens*. COREQ is a 32-item checklist for interviews and focus groups, and was developed for "explicit and comprehensive reporting of qualitative studies"(p. 1). There are three domains: research team and reflexivity, study design, and data analysis and reporting.

| **Table 1**  *Consolidated criteria for reporting qualitative studies: 32-item checklist* | | | |
| --- | --- | --- | --- |
| No Item | Guide question / description | Yes, information included | No, information not included |
| **Domain 1: Research team^[[1]](#footnote-1)^** |  |  |  |
| Personal Characteristics |  |  |  |
| 1. Interviewer / facilitator | Which author/s conducted the interview or focus group? | X |  |
| 1. Credentials | What were the researcher’s credentials? E.g. PhD, MD | X |  |
| 1. Occupation | What was their occupation at the time of the study? | X |  |
| 1. Gender | Was the researcher male or female? | X |  |
| 1. Experience and training | What experience or training did the researcher have? | X |  |
| Relationship with participants |  |  |  |
| 1. Relationship established | Was a relationship established prior to study commencement? | X |  |
| 1. Participant knowledge of the interviewer | What did the participants know about the researcher? e.g. personal goals, reasons for doing the research | X |  |
| 1. Interviewer characteristics | What characteristics were reported about the interviewer/facilitator? e.g. Bias, assumptions, reasons and interests in the research topic | X |  |
|  |  |  |  |
|  |  |  |  |
| **Domain 2: study design** |  |  |  |
| Theoretical framework |  |  |  |
| 1. Methodological orientation and theory | What methodological orientation was stated to underpin the study? e.g. grounded theory, discourse analysis, ethnography, phenomenology, content analysis | X |  |
| Participant selection |  |  |  |
| 1. Sampling | How were participants selected? e.g. purposive, convenience, consecutive, snowball | X |  |
| 1. Method of approah | How were participants approached? e.g. face-to-face, telephone, mail, email | X |  |
| 1. Sample size | How many participants were in the study? | X |  |
| 1. Non-participation | How many people refused to participate or dropped out? Reasons? | X |  |
| Setting |  |  |  |
| 1. Setting of data collection | Where was the data collected? e.g. home, clinic, workplace | X |  |
| 1. Presence of non-participants | Was anyone else present besides the participants and researchers? | X |  |
| 1. Description of sample | What are the important characteristics of the sample? e.g. demographic data, date | X |  |
| Data collection |  |  |  |
| 1. Interview guide | Were questions, prompts, guides provided by the authors? Was it pilot tested? | X |  |
| 1. Repeat interviews | Were repeat interviews carried out? If yes, how many? | X |  |
| 1. Audio / visual recording | Did the research use audio or visual recording to collect the data? | X |  |
| 1. Field notes | Were field notes made during and/or after the interview or focus group? | X |  |
| 1. Duration | What was the duration of the interviews or focus group? | X |  |
| 1. Data saturation | Was data saturation discussed? | X |  |
| 1. Transcripts returned | Were transcripts returned to participants for comment and/or correction? | X |  |
| **Domain 3: analysis and findings**  Data analysis |  |  |  |
| 1. Number of data coders | How many data coders coded the data? | X |  |
| 1. Description of the coding tree | Did authors provide a description of the coding tree? | X |  |
| 1. Derivation of themes | Were themes identified in advance or derived from the data? | X |  |
| 1. Software | What software, if applicable, was used to manage the data? | X |  |
| 1. Participant checking | Did participants provide feedback on the findings? | X |  |
| Reporting |  |  |  |
| 1. Quotations presented | Were participant quotations presented to illustrate the themes / findings? Was each quotation identified? e.g. participant number | X |  |
| 1. Data and findings consistent | Was there consistency between the data presented and the findings? | X |  |
| 1. Clarity of major themes | Were major themes clearly presented in the findings? | X |  |
| 1. Clarity of minor themes | Is there a description of diverse cases or discussion of minor themes? | X |  |
| *Note:* This table is based on the original checklist in Tong and colleagues’s (2007) article: *Consolidated criteria for reporting qualitative research (COREQ): a 32-item checklist for interviews and focus groups.* | | | |

**References**

Tong, A., Sainsbury, P., & Craig, J. (2007). Consolidated criteria for reporting qualitative research (COREQ): a 32-item checklist for interviews and focus groups. *International Journal for Quality in Health Care, 19*(6), 349-357. doi:10.1093/intqhc/mzm042

1. In our paper, we only have "Research team" and do not include "and refelxitivity", since we did not find the latter relevant. [↑](#footnote-ref-1)
