# Supplementary material for: Qualitative evaluation of the implementation of “Tuning in to Kids” in Norwegian Kindergartens
Source: BMC Psychol. 2023 Mar 30;11:87. doi: 10.1186/s40359-023-01088-4 (PMC10060915; doi:10.1186/s40359-023-01088-4)
Supplement: Supplementary file 2 — Additional file 2. Table 1. All codes Time 1. Table 2. All codes Time 2. Table 3. Code groups. Table 4. Themes. [file 40359_2023_1088_MOESM2_ESM.docx]

Table 1

*All codes Time 1*

| 1. When employees learn and see the effect on children, they will become interested in doing the tasks as good as possible (1) |
| --- |
| 1. Research based methods that seem good for the children will initiate interest to do imposed tasks (2) / Research based knowledge is a reason as to why one should do something (3) / We will work with this because it is based on research (4) |
| 1. It is good to develop and to learn things one knows is good (5) / The majority are happy to learn new things and to get the chance to develop (6) / Every person must work with himself/herself and make personal goals (7) Nice when those who have worked in a kindergarten for a long time discover that there are things to learn (8) |
| 1. This is familiar (9) / This is familiar, but now it is put in a system and different words are used (10) / The theory is familiar, but now we learn how we should do it (11) / The theory is familiar (12) / It is a good thing to be familiar with the things we are going to work with (13) / This is familiar, but it is put in a system and used different words (14) |
| 1. It will be motivating if it seems meaningful and if one notice effect (15) |
| 1. Processes of change asks something of everyone (16) / We have gotten tools and it is the entire division’s task to implement it (17) |
| 1. Important with management to ensure that all levels are reached and that it is systematical for it to become implemented (18) / We work on something that is common, across levels of workmanship (19) |
| 1. Frustrating when what one thinks is received and understood is still not executed (20) |
| 1. Execution is connected to a busy workday (21) |
| 1. Those who are passionate about it are motivating (22) |
| 1. If everyone understands why, then it is all right to change the ways of working (23) / You need to create a good understanding with the others, if not there will definitely not be a change (24) / Understanding is important to comprehend the entirety about why one ought to do the things one are to do (25) / Those who don’t understand, struggle when it comes to pass on information(26) / Information is crucial for motivation (27) / Without information we cannot make progress (28) / Need to know what something is before it can be passed on and be motivated to (29) / Looses something in the process of passing on information (30) |
| 1. It is important that everyone is aware of different ways of working and of what will happen when change is accomplished (31) |
| 1. The main motivation is to make it as good as possible for the children (32) / We wish the best for the children (33) / We will work with this for the best for the children (34) |
| 1. Direct feedback to employees in the workday has very good effect (35) |
| 1. Time spent is the challenge (36) / Challenging to have enough time in the kindergarten (37) / It is important with a lot of time to implementation (38) / The greatest impediment to accomplish something in a kindergarten little time with all employees (39) |
| 1. It was important to have a course with the entire staff. Passing on information is difficult (40) / Same information, same basis to work from (41) |
| 1. As long as one manages it most of the time (42) |
| 1. Concrete to do, easy to connect to practice, it works, and it is reasonable (43) / The kindergarten has been missing something so concrete (44) |
| 1. Knowledge motivates me, it must have a meaning (45) / I think the workday becomes a little brighter when I learn new things (46) / My interest for the field makes me interested in the intervention (47) |
| 1. What works for me, works for others is how I think (48) |
| 1. With something being important, smart, and good, as well as one can see that it works, those are good reasons to why one ought to do something (49) |
| 1. How one experience to perform imposed tasks, depends on the results of doing them (50) |
| 1. Looking forward to share with the entire staff (51) / Excited and looking forward to it, the other ones seem so pleased (52) |
| 1. I like to be up to date and to have order (53) |
| 1. Change is slow and is influenced by what you have experienced in the past (54) / This is how we have always done it and it works fine, so then we keep on like this (55) / It takes quite strong motivation, and that one sees effect, in order to attain change (56) |
| 1. Repetition is important to not go back to old patterns (57) |
| 1. Talk about what is to be implemented together (58) / If it is not said, then it disappears in the many work tasks (59) |
| 1. Not too busy to not do the job the way it is supposed to be done (60) / It is their job (61) |
| 1. This is exciting and I like my subject, want to develop (62) |
| 1. Statistics over people in organization (63) |
| 1. Statistics of what awaits and where one is at (64) / To know the statistic and to use knowledge from it (65) |
| 1. Experience boundaries (66) / Get to know oneself is positive (67) |
| 1. No matter what I come up with, they go like: "Yes, yes, yes!" (68) |
| 1. Important to connect with the person where she/he is at (69) |
| 1. Important that everyone tries, in a big project demanding resources and based on research (70) |
| 1. Perhaps I have something to learn from the others (71) |
| 1. I am familiar with what it takes (72) |
| 1. To get some of that aha-experience is quite important (73) / Aha-experience (74) |
| 1. I want the others shall experience the joy of learning something new (75) |
| 1. They need to feel like this is something they own (76) / To feel like one is included and is important in all functions (77) |
| 1. There is always an overall expectation to keep up with the times (78) |
| 1. Theory is one thing, and practice something else (79) / The million-dollar question is why the understanding sort of may be present and even so, it is difficult to see change in practice (80) |
| 1. The workday in the kindergarten is not always as we plan it to be (81) |
| 1. My challenge is to find the meeting point (82) / Much to do, little time for meetings (83) / Few meeting points (84) |
| 1. When it comes from FUS, then we shall be loyal towards FUS (85) |
| 1. Very interesting and exciting (program/intervention) (86) |
| 1. Good that FUS does this because it is so important (87) / FUS is going for this, and research is the basis (88) |
| 1. Everyone is very positive to get going with this intervention (89) |
| 1. A foundation to keep building on (90) |
| 1. We are good at doing things we are supposed to do, and we get information about it (91) |
| 1. It is important that everyone in the kindergarten have the same information. Then we have the same knowledge and can work from the same starting point (92) / If we do the same, then we stand stronger together (93) |
| 1. Adds concretization and measurement for shared work (94) |
| 1. "Oh, what is new now?" (95) |
| 1. From "Oh!" via introduction to positivity (96) / An effort with something new, but when introduced to the intervention the enthusiasm and interest awakes (97) |
| 1. The experience of one’s own control vary with the function in the kindergarten (98) |
| 1. With less education the words may be difficult and thoughts about the intervention being difficult may appear (99) |
| 1. Scary to be paid attention to (100) |
| 1. Guidance and clear expectations are important when starting up with a new intervention (101) / What is most important is that we go on and show that this is important (102) / "This is what we shall be working on in the time to come" (103) |
| 1. It takes a great amount of energy to follow up on practice of change over time (104) |
| 1. The possibility to really dive into it, get a grip on it and manage to continue with it (105) |
| 1. Acceptable processes when it comes to understanding and time (106) |

Table 2

*All codes Time 2*

| 1. We work on something that is mutual for us, on different professional levels (107) |
| --- |
| 1. Can be challenging to include the leaders (108) |
| 1. It’s experienced as effective and challenging (109) |
| 1. I’m not perfect and not everything can be solved with smooth TIK (110) / Every now and then is good enough (111) |
| 1. We don’t reach the “finish line”, but can’t let go yet (112) |
| 1. It’s too natural that it diverts too quickly (113) |
| 1. I can’t choose arbitrarily what I want to be doing (114) |
| 1. One must make one’s own, one’s own emphatical sounds, then it’s good (115) |
| 1. You have the theory, but you haven’t quite rehearsed on the practice (116) |
| 1. It’s a different way to do it, and it’s det TIK-way (117) 2. Understand that this is the best for the children (118) |
| 1. Challenging to turn the entire practice (119) |
| 1. Fell a bit away after a period of closed kindergartens (120) / Difficult to accomplish with home office (121) / Difficult to be in a relation when not meeting (122) / Something disappears when we don’t meet (123) / Difficult to supervise as long as there is cohorts and much time spent outside (127) |
| 1. The corona period was much work, but inner worry (128) |
| 1. Effective and concrete, I’ve become very found of TIK (129) / It’s something concrete, something I can point out (130) / The kindergarten have been missing something this concrete (131) |
| 1. Everyone was there and got to hear the same (132) / Everyone has gone through this (133) / We must do it the same way (134) / Everyone was “on the same ship” right away (135) |
| 1. The material worked out for everyone (136) |
| 1. Looses something in the process of passing on information (137) |
| 1. Understands what it’s about (138) / Why TIK is important, why it’s important to acknowledge children’s feelings (139) / “Now we get it, now we get what the other lady said” (140) / So that we shall understand (141) / Include everyone. Why do we do this? (142) |
| 1. The use of it varies (143) |
| 1. A good foundation plus repetition (144) |
| 1. Not emptying all staff from one group (145) |
| 1. Focus somewhere else (146) / Different focus (147) / Different focus (148) |
| 1. It must be implemented well (149) |
| 1. I feel I’ve got control on what I do during my workday, we have a plan as for what we shall do and what we shall accomplish (150) |
| 1. TIK has added something important and it’s important to me to pass that on to everyone (151) |
| 1. “Tune” in on where the child is at (152) |
| 1. Take care of the child (153) |
| 1. Aha experience (154) / Aha experience (155) |
| 1. I’m very excited (156) |
| 1. Feel it on my body (157) |
| 1. We’re shall manage to prioritize this project (158) |
| 1. See that it works (159) |
| 1. Someone needs to adjust their attitude a little (160) |
| 1. “Do you remember, that hand…” (161) / The five steps (162) |
| 1. I think supervision in the moment is very effective (163) |
| 1. The culture to supervise people in the moment is dependent on the person (164) |
| 1. Feedback to colleagues isn’t about corrections, but about reminders (165) |
| 1. We have a good foundation (166) |
| 1. Thoroughly training was positive (167) |
| 1. Much to do, little time for meetings (168) / Few meeting points (169) |
| 1. Being close on and more time after the corona restrictions (170) / That’s exactly what it became: “Tuning in to kids” (171) / I could lower my shoulders (172) / So then it was really TIK! (173) / “You are to be here and that’s your job today” was a notable consequence from shorter opening hours and stricter guidelines (174) |
| 1. The opposite of TIK in relation to those I was not with (175) |
| 1. It wasn’t as good when it wasn’t Sophie! (176) |
| 1. Explosive progression (177) |
| 1. The springtime was odd (178) |
| 1. We had role play in front of the parents! (179) |
| 1. Lost control a little when it didn’t go as expected (180) |
| 1. We felt pretty “green” ourselves! (181) |
| 1. It was a good feeling (182) |
| 1. “I managed to develop the staff, kind of”. Sense of mastery (183) |
| 1. Felt they agreed (184) |
| 1. Important to really get a hang on it (185) |
| 1. Keep it engaging/maintained (186) / Repetition to keep it engaging/maintained (187) |
| 1. It’s often most engaging in the beginning (188) |
| 1. This is too important to let it fade away! (189) |
| 1. Getting trapped (190) |
| 1. To give clues keeps it engaging (191) |
| 1. I’m impressed by my colleagues (192) |
| 1. Important with video when we don’t have Sophie (193) |
| 1. “Very important that we were two people that were present to listen to Sophie, that it wasn’t just me” (194) |
| 1. Important with supervision (195) |
| 1. They were good at sharing which “traps” they had gotten into (196) |
| 1. We are always scattered (197) |
| 1. There’s something in prioritizing (198) |
| 1. It keeps on coming new stuff out of the blue (corona related, 199) |
| 1. Everyone thinks about it, but no one knows what will happen (corona related, 200) |
| 1. Enough time is important (201) / Enough time to establish and to question (202) / Too little time to focus on what we actually are supposed to do (203) / Rather spend enough time to get a hang of it. Then it doesn’t seem like there is as much too loose, the stakes are not too high (204) |
| 1. Based on varying education, someone thinks it’s exciting and interesting, while others think it’s boring and difficult to understand (205) |
| 1. To get to know oneself is positive (206) |
| 1. What is most important is that we go on and show that this is important (207) / "This is what we shall be working on in the time to come" (208) |
| 1. If it’s not communicated, it will disappear in many work tasks (209) |
| 1. Positive to focus on TIK since FUS is going for this (210) / We shall go for it (211) / This is what we do now, and this is what FUS is going for (212) |
| 1. Still positive, but not as planned (213) |
| 1. It was a difficult job to sort out what was CLASS and what was TIK, it’s a mix in my head. Huge to be a part of (214) |
| 1. “Make-the-days-go-around”-kind of activity (215) |
| 1. Easy to think the thought, not as easy in practice (216) |
| 1. If I only could have had supervisions… (217) |
| 1. Choosing to have department meeting is not for the best for the children (218) |
| 1. It’s done differently at the departments, which leads to challenges when it comes to changing department (219) |
| 1. A whole, full-scale package! (220) |
| 1. Already familiar, different presentation and words (221) |
| 1. Things matures a little (222) |

Table 3

*Code groups*

| Time 1 | Time 2 |
| --- | --- |
| 1. Based on research | 1. Not perfect |
| 1. Personal interest, development | 1. Everyone meets and knows the same |
| 1. Understand – the same information – meet with each other | 1. Concrete |
| 1. Familiar, not new | 1. To understand, master / aha-experience |
| 1. Enough time, difficult to change what is a habit | 1. Where is the focus |
| 1. The best for the children/common goal? | 1. Exemplary / role model |

Table 4

*Themes*

| The scientific aspect  “The zone of proximal development” | **Understanding the rationale** |
| --- | --- |
| The aspect of experiences  Aha experience | **Aha experiences** |
| The professional aspect  The challenges / research-to-practice gap | **The research-to-practice gap** |
| The personal aspect | **The main motivation** |
